# A training plan to implement lung ultrasound for diagnosing pneumonia in children

**DOI:** 10.1038/s41390-021-01928-2

**Published:** 2021-12-30

**Authors:** Carmina Guitart, Esther Esteban, Judit Becerra, Javier Rodríguez-Fanjul, Francisco José Cambra, Mònica Balaguer, Iolanda Jordan

**Affiliations:** 1 Pediatric Intensive Care Unit, Hospital Sant Joan de Déu, University of Barcelona, Barcelona, Spain; 2 Immunological and Respiratory Disorders in the Pediatric Critical Patient Research Group, Institut de Recerca Sant Joan de Déu, University of Barcelona, Barcelona, Spain; 3 Section of Zoology and Biological Anthropology, Department of Evolutionary Biology, Ecology and Environmental Sciences, Faculty of Biology, Universitat de Barcelona, Barcelona, Spain; 4 Institut de Recerca de la Biodiversitat (IRBio), Universitat de Barcelona, Barcelona, Spain; 5 Neonatal Intensive Care Unit, Department of Pediatrics, Hospital Germans Trias i Pujol, Autonomous University of Barcelona, Badalona, Spain; 6 Pediatric Infectious Diseases Research Group, Institut de Recerca Sant Joan de Déu, CIBERESP, Barcelona, Spain

## Abstract

**Background:**

Lung ultrasound (LUS) for critical patients requires trained operators to perform them, though little information exists on the level of training required for independent practice. The aims were to implement a training plan for diagnosing pneumonia using LUS and to analyze the inter-observer agreement between senior radiologists (SRs) and pediatric intensive care physicians (PICPs).

**Methods:**

Prospective longitudinal and interventional study conducted in the Pediatric Intensive Care Unit of a tertiary hospital. Following a theoretical and practical training plan regarding diagnosing pneumonia using LUS, the concordance between SRs and the PICPs on their LUS reports was analyzed.

**Results:**

Nine PICPs were trained and tested on both theoretical and practical LUS knowledge. The mean exam mark was 13.5/15. To evaluate inter-observer agreement, a total of 483 LUS were performed. For interstitial syndrome, the global Kappa coefficient (*K*) was 0.51 (95% CI 0.43–0.58). Regarding the presence of consolidation, *K* was 0.67 (95% CI 0.53–0.78), and for the consolidation pattern, *K* was 0.82 (95% CI 0.79–0.85), showing almost perfect agreement.

**Conclusions:**

Our training plan allowed PICPs to independently perform LUS and might improve pneumonia diagnosis. We found a high inter-observer agreement between PICPs and SRs in detecting the presence and type of consolidation on LUS.

**Impact:**

- Lung ultrasound (LUS) has been proposed as an alternative to diagnose pneumonia in children. However, the adoption of LUS in clinical practice has been slow, and it is not yet included in general clinical guidelines.
- The results of this study show that the implementation of a LUS training program may improve pneumonia diagnosis in critically ill patients. The training program’s design, implementation, and evaluation are described. The high inter-observer agreement between LUS reports from the physicians trained and expert radiologists encourage the use of LUS not only for pneumonia diagnosis, but also for discerning bacterial and viral patterns.

## Introduction

Point-of-care (POC) lung ultrasound (LUS) has come to stay as a solid alternative to traditional radiological diagnosis in pediatric patients. LUS in critically ill patients streamlines the diagnostic process since the clinicians themselves perform and interpret the image. POC-LUS contributes to early diagnoses and thus improves the quality of care.^1–4^ It is a quick and radiation-free procedure that can be easily introduced in different pediatric departments because ultrasound devices are increasingly smaller, more user-friendly, and provide high-quality images.^5^

Respiratory infections represent, depending on the time of the year, between 30 and 70% of the total admissions in pediatric intensive care units (PICUs). These percentages would represent about 150–200 patients per year in our unit. The etiology is viral in 60–70% of the cases and depending on the etiological agent involved, the treatment may include antibiotics, corticosteroids, isolation, and other therapies. In the case of pneumonia, making a differential diagnosis between a viral and bacterial infection can be complex. The clinical symptomatology is nonspecific and although chest X-ray (CXR) is considered the best diagnostic option in children, it shows low specificity as regards the diagnosis of a bacterial etiology.^6^

POCUS (point-of-care ultrasound) is increasingly being utilized in neonatal and pediatric critical care as a valuable adjunct to clinical examination.^2^ It involves a focused assessment and provides anatomical and/or physiological information that can be integrated with clinical and laboratory data, making timely and accurate decisions possible. Following POCUS guidelines may help in standardizing clinical practices in acute care settings. POC-LUS is helpful to semi-quantitatively evaluate lung interstitial syndrome and to detect pneumonia and pleural effusions in infants and children.^2^ Radioprotection, manageability, and a more targeted use of image tests, when performed by the physician in charge of the patient, are some of the most important advantages LUS has over CXR.^2,4,5^

LUS allows for a more sensitive and specific diagnosis of pneumonia, earlier than chest X-rays. It also decreases radiation exposure by up to 30–60%^3,6^ because it reduces the total CXR use, the number of CXRs performed, and the mean dose received per patient. The fact that pneumonia shows consistent ultrasound images in children of different ages underlines that its diagnostic accuracy is well beyond that of conventional radiology.^7–10^ However, the use of ultrasound for the diagnosis of lung infections is an operator-dependent skill, and adequate training is required for effective clinical use.^11^ There are some recommendations on how to perform a LUS examination, mainly in adults.^1,12,13^ Recent meta-analyses summarize the use of LUS in different pediatric interventions,^4,14^ but the studies performed to date are few in number^15^ and as far as we know, a limited number of them are focused on pneumonia. Moreover, although training should be structured and taught by LUS experts who have the necessary background, knowledge, and experience, there is little information regarding the level of training required for independent practice^12^ by PICPs.

Some systematic reviews conclude that even though LUS seems to be a promising tool for diagnosing pneumonia in children, the high level of heterogeneity across individual studies and the absence of a reliable reference standard make the findings questionable, indicating that more methodologically rigorous studies are needed.^10,16^

We hypothesized that the implementation and standardization of a theoretical and practical training plan for pneumonia using LUS can be readily used to teach PICPs.

The second objective was to analyze the inter-observer agreement between senior radiologists (SRs) and PICPs as regards a final diagnosis of pneumonia using LUS.

## Methods

This was a prospective longitudinal and interventional study conducted at the Hospital Sant Joan de Déu PICU. It was the first part of a clinical trial (PROcalcitonin and Lung UltraSound algorithm to diagnose severe Pneumonia in critical pediatric patients [PROLUSP study]; trial registration number: NCT04217980).^17^ The training plan was carried out from January 2017 to April 2017. The recruitment period and follow-up took place afterward, from September 2017 to December 2019.

For the successful implementation of LUS for pneumonia diagnosis, we adopted a two-phase approach: the first phase was intended to implement a training plan to teach PICPs how to diagnose pneumonia using LUS. The second phase was defined to evaluate that training and to analyze the agreement regarding LUS diagnosis between radiologists and the PICPs trained.

### First phase: training plan

This first phase was carried out in three steps. The first step was to identify internal and external PICU challenges, focusing on both short and long-term requirements. At this point, the possible difficulties in implementing the plan were determined to be: (i) the availability of the ultrasound machine to perform the LUS bedside at any time (as per the research authorities’ recommendation, only one ultrasound device was exclusively dedicated to the clinical trial), and (ii) the training difficulties during the working day due to the day-to-day clinical responsibilities.

The second step was to design the training plan. The training knowledge and skills to be developed were identified as (i) to know the general concepts of echography, (ii) to learn how to use an ultrasound device in general and how to use it specifically for the clinical trial, (iii) to learn how to evaluate and understand normal ultrasound patterns, and (iv) to learn the established classification of pathologies based on ultrasound patterns.

Regarding the specific LUS device, it was a portable ultrasound machine (Toshiba Xario 100) with a 12-MHz linear or a 5-MHz convex probe that could be used depending on the weight or size of the patient. The scans to be systematically taken were defined as three areas for each hemithorax (anterior, lateral, and posterior) according to the international recommendations.^12^ Each area had to be examined longitudinally and transversely.

**Fig. 1:**
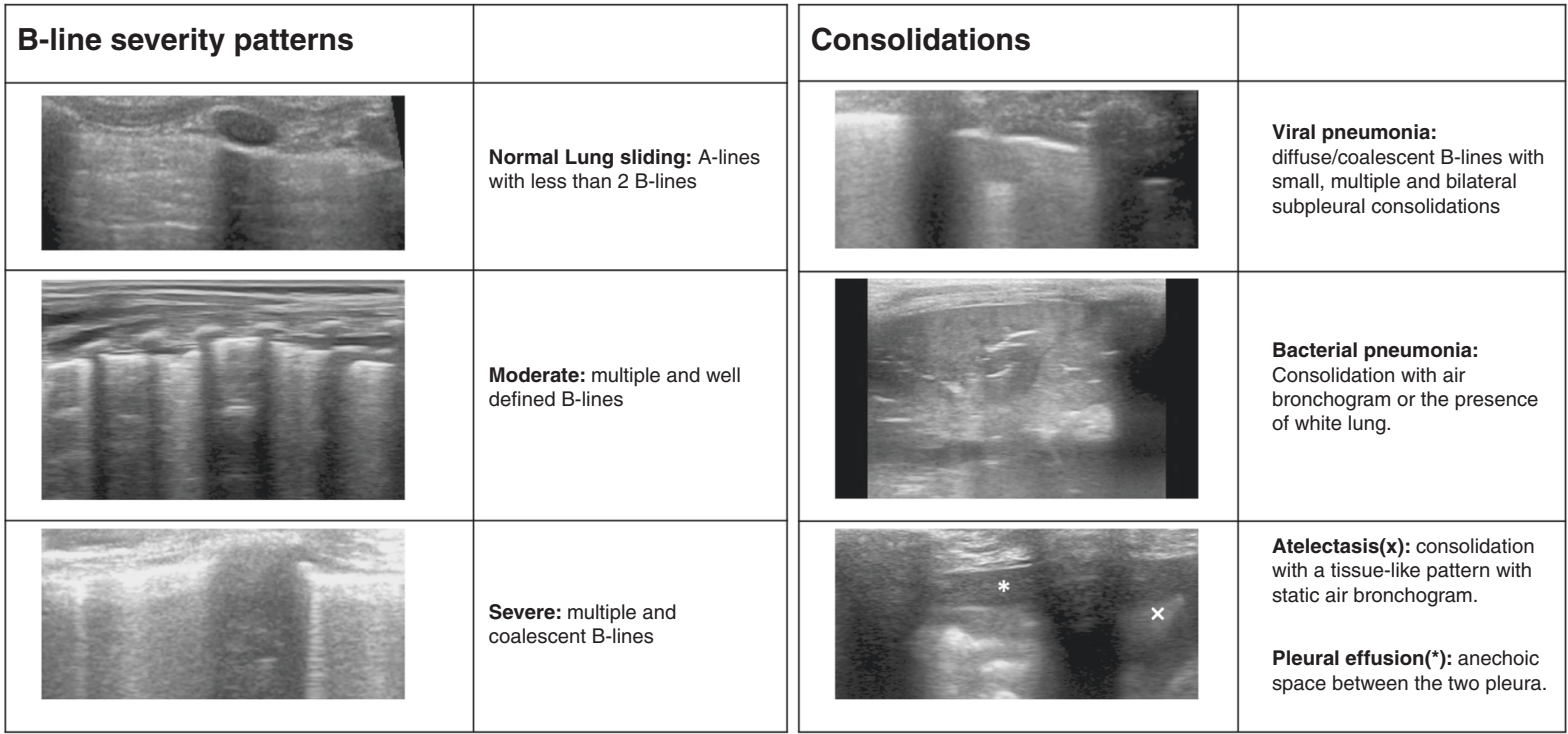
Images of lung ultrasound interstitial syndrome and consolidation patterns. Lung ultrasound B-line/interstitial syndrome severity and consolidation patterns.

Based on the “International Evidence-Based Recommendations for Point-of-care Lung Ultrasound”,^12^ the different characteristic patterns to be taught were also defined. In each area, the following needed to be evaluated:^12^ A-lines, B-lines (number and distance between them), lung sliding (M-mode), pleural space, lung consolidations, small subpleural consolidations, dynamic air bronchogram, vascular pattern, presence of lung point, and lung pulse. The determination of a bacterial pneumonia ultrasound pattern was based on the presence of lung consolidation with air bronchograms, which in the initial stages is detected as small subpleural hypoechoic zones of more than 1 cm with air bronchogram (not seen using conventional CXR).^18–20^ The determination of a viral pneumonia ultrasound pattern was based on the presence of coalescent B-lines with small subpleural consolidations of <1 cm, without an air bronchogram.^21,22^ Figure 1 shows the different possible interstitial syndrome and consolidation patterns.

The third step was to develop the training program, considering the following aspects: training content, target population, number of physicians, schedule, duration, and place of training. The training plan was carried out over the three following months. It was organized by four experienced senior physicians (authors of the manuscript), who used general critical care lung ultrasonography regularly. They have more than 10 years of ultrasound experience (30–50 lung scans per month) and they are teachers and trainers for thoracic ultrasound learning courses. More details regarding their experience can be found in Table 1.

**Table 1:** PICU and LUS experience of trainer physicians.

	Age	PICUe	LUSe	LUS/month
Trainer 1	45 y	13	5–10	30–40
Trainer 2	47 y	16	5–10	30–40
Trainer 3	37 y	7	5–10	30–40
Trainer 4	40 y	10	5–10	30–40

*Y* years old, *PICUe* years of experience of the trainer physicians in PICU, *LUSe* years of experience of the trainer physicians in LUS.

The training was divided into theoretical and practical parts. The theoretical part was done through a virtual platform. It was divided into three units: (1) general concepts in ultrasound (basic ultrasonography knowledge, equipment and terminology, elementary images, and artefacts), (2) the normal LUS pattern, and (3) pathological LUS patterns. It contained protocols, algorithms, interactive exercises, and videos of clinical cases. A theoretical exam with fifteen questions had to be taken before and after the theoretical training. This exam is attached as supplementary material. The practical part was carried out with the physician under training performing bedside LUS while being formally supervised by one of the senior physicians. It was determined that each operator should perform a minimum of thirty lung ultrasounds before the practical exam. The practical training was evaluated and scored by one experienced physician. The items to be described and the marks were defined by the four experienced trainer physicians, including the following: choosing the correct probe for the patient’s age and weight, scanning all the thorax areas defined, and writing a proper report with all the characteristics found.

Over the course of the study, team sessions focusing on the diagnosis of pneumonia with LUS were repeated every 3 months to ensure quality and consistency in the LUS exam. The supplemental data included in the Shah et al. article was used.^23^

### Second phase

#### Training evaluation

Both a written and practical test were administered to evaluate the LUS skills acquired. Participants needed to get a minimum score of 11/15 on each test in order to pass the final exam. A satisfaction survey to evaluate the training plan was also given to the participating physicians. The exam and the satisfaction survey are included as supplementary materials.

The inter-observer agreement study was done after patient recruitment for the clinical trial ended in December 2019. LUS and CXR were performed on any patient with suspected pneumonia who was admitted to the PICU during the clinical trial period. Patients were sampled consecutively. Three LUS were done for each patient. The first LUS was performed when the pneumonia was initially suspected, at the same time as the CXR. The physicians performing the LUS were blinded to the CXR and to the lab results. The other two LUS were carried out during the five following days in order to monitor LUS evolution. The second LUS was done between the 2nd and 3rd day, and the third LUS between the 4th and 5th day. These timings were established to be able to better detect interstitial syndrome or consolidation pattern changes or any complications over the course of the patient’s clinical evolution. All the LUS images were saved. Intensive care physicians who had received special training in LUS and had at least 3 years of experience oversaw the quality of each acquired image. Clinical results were recorded in the patient’s clinical history. Both observers, the physicians in training and the senior radiologist, blinded to each other’s results, analyzed LUS and CXR images and handed in their results report, which was also recorded in the patient’s clinical history. The senior consulting radiologist was a radiologist who specializes in pediatrics and has dedicated his entire 15-year career to this field, with a special focus on pediatric pulmonary pathologies. Written parental informed consent was mandatory. A flow diagram is provided as supplementary material.

#### LUS agreement

Afterwards, the inter-observer agreement (measured by Cohen’s Kappa) between the PICU sonologists’ interpretation of LUS results and the radiologists’ interpretation of LUS results was analyzed. The concordance was evaluated for all items recorded: the grade of interstitial syndrome, the presence and type of consolidation, and the presence of pneumothorax and/or pleural effusion. To analyze whether there was an improved agreement with the radiologists as the study progressed, the global concordance for each tercile was calculated. A basic learning curve was obtained.

The final diagnosis was established by a clinical expert and was characterized based on the CDC’s definition of pneumonia:^24^ compatible clinical symptoms (fever, cough, tachypnea, shortness of breath, abnormal respiratory auscultation sounds, hypoventilation, tubular breath sounds), compatible chest X-ray (CXR) (lobar consolidation, airspace opacity, pleural effusion, bullae, etc.) and blood test abnormalities with leukocytosis, neutrophilia, a C-reactive protein higher than 50 mg/L, and/or a procalcitonin level higher than 1 ng/mL.

### Outcomes

The primary outcome targeted was the inter-observer agreement, as measured by Cohen’s Kappa, between the PICU sonologists’ interpretation of LUS and CXR results versus the radiologists’ interpretation of LUS and CXR results.

The secondary outcome assessed was the evaluation of the progress of the LUS training plan and its efficacy.

### Statistical analysis

Qualitative variables were described as absolute and relative counts, while quantitative variables were summarized as mean ± standard deviation or as median and interquartile range (IQR), depending on the normality of the variable.

For the inter-observer agreement study, a contingency table was generated for each visit, which included the counts from the cross-assessments by the physicians in training and the radiologist. The concordance level was assessed with the Kappa coefficient (*K*)^25^ and the weighted Kappa in the case of ordinal variables. The coefficients were estimated using the R package *psych*. The value of *K* was interpreted according to the following scale: <0 poor, 0.01–0.2 slight, 0.21–0.40 fair, 0.41–0.6 moderate, 0.61–0.8 substantial, 0.81–1.00 almost perfect.^26^ The Kappa coefficients of the three visits were compared using a permutation test. When the equality hypothesis was not rejected, an overall *K* across visits was estimated using the method reported in King et al.^27,28^ This overall Kappa was estimated using the R package *cccrm.*^29^ All the analyses were done using R v3.6.3.^30^

## Results

### First phase: LUS training plan

In our PICU, only 4 of the 13 staff physicians had previously received accredited formal training on performing LUS and interpreting LUS findings. Therefore, according to the PICU requirements, nine physicians needed to be trained and tested in both theoretical and practical LUS knowledge. After the training program, all the participants were evaluated and performed well. The mean mark for the exam was 13.5/15. The satisfaction questionnaire for the training plan, completed by the physicians who participated, resulted in an average score of 4.7 out of 5 points.

### Second phase: training evaluation and agreement analysis

A total of 254 children were assessed for study eligibility and 194 were ultimately enrolled. The patients’ characteristics are shown in Table 2. A total of 483 LUS were performed. Of these, 190 LUS were done during the baseline visit, 164 LUS at the second visit, and 129 LUS during the third visit. The LUS operator performed the 3 scans for the same patients. When this was not possible due to them not being present in the clinic at that time, the images were reviewed, analyzed, and reported by the same LUS operator afterward.

**Table 2:** Demographic and clinical variables.

	Total (*N* = 194)
Gender (male), *n* (%)	81 (41.8)
Age (days), median (IQR)	134 (39–554)
Weight (kg), median (IQR)	6.30 (4.3–11.0)
Comorbidities, *n* (%)	
None	116 (59.8)
Respiratory	31 (16.0)
Cardiovascular	15 (7.7)
Infectious diseases	2 (1.0)
Neurological	7 (3.6)
Hematology–oncology	2 (1.0)
Other	21 (10.8)
Reason for admission, *n* (%)	
Infection^a^	149 (76.8)
Trauma	7 (3.61)
CV surgery/heart failure	9 (4.64)
Surgery (Abd, trauma, NS)	4 (2.06)
Other	25 (12.9)
Severity upon admission	
PRISM, median (IQR)	2 (0–5)
Length of stay	
PICU (days), median (IQR)	7.0 (4.0–14.7)
Hospitalization (days), median (IQR)	26.0 (17.5–43.5)
Respiratory support	
HFNC, *n* (%)	103 (53.1)
NIV, *n* (%)	166 (85.6%)
CMV, *n* (%)	85 (43.8)
NO, *n* (%)	9 (4.6)
Inotropic support, *n* (%)	23 (11.9)
ECMO, *n* (%)	3 (1.6)
Death, *n* (%)	1 (0.52)
Leukocytes, median (IQR)	10,800 (7500–15,575)
CRP mg/L, median (IQR)	43.1 (20.0–96.1)
PCT ng/mL, median (IQR)	0.60 (0.18–2.26)
Antibiotic therapy, *n* (%)	143 (73.7)

*IQR* interquartile range, *CV* cardiovascular, *Abd* abdominal surgery, *NS* neurosurgery, *PRISM* pediatric risk of mortality, *PICU* pediatric intensive care unit, *HFNC* high flow nasal cannula, *NIV* non-invasive ventilation, *CMV* conventional mechanical ventilation, *NO* nitric oxide, *ECMO* extracorporeal membrane oxygenation, *CRP* C-reactive protein, *PCT* procalcitonin.

^a^Infection: meningitis, pneumonia, sepsis, bronchiolitis.

As regards to B-line/interstitial syndrome severity, the inter-observer agreement between the trainee PICU sonologists and radiologists showed a Cohen’s Kappa of 0.51 (95% CI 0.43–0.58), which is interpreted as moderate agreement. Most discrepancies occurred between single B-lines vs. confluent B-lines. As for the presence of consolidation, the overall *K* was 0.67 (95% CI 0.53–0.78), implying substantial agreement. With respect to the consolidation pattern results, the overall *K* was 0.82 (95% CI 0.79–0.85), which is interpreted as almost perfect agreement. The overall *K* for the presence of pleural effusion was 0.78 (95% CI 0.66–0.86), implying substantial agreement. Regarding the presence of pneumothorax, there was only one discrepancy. However, due to the small sample of pneumothorax cases, the *K* estimate yielded a low value of 0.15. The result is not meaningful due to the low prevalence and few cases of pneumothorax in our study. More details are shown in Table 3.

**Table 3:** Agreement results between the radiologist’s and trained physicians’ LUS report for each finding.

LUS findings	Kappa coefficient (95% CI)				
	Baseline visit	Second visit	Third visit	Overall *K*	*p*-value
Interstitial syndrome severity	0.56 (0.41–0.69)	0.55 (0.36–0.71)	0.39 (0.15–0.58)	0.51 (0.43–0.58)	0.333
Consolidation	0.59 (0.37–0.81)	0.73 (0.53–0.94)	0.55 (0.32–0.77)	0.67 (0.53–0.78)	0.827
Consolidation pattern	0.79 (0.71–0.86)	0.80 (0.73–0.88)	0.83 (0.75–0.90)	0.82 (0.79–0.85)	0.842
Pleural effusion	0.69 (0.52–0.87)	0.81 (0.65–0.97)	0.44 (0.16–0.71)	0.78 (0.66–0.86)	0.161
Pneumothorax	0.00 (0.00–0.00)	0.66 (0.05–1.0)	1.00 (1.00–1.0)	0.15 (0.09–0.2)	0.242

Kappa value < 0: poor, Kappa value 0.01–0.2: slight, Kappa value 0.21–0.40: fair, Kappa value 0.41–0.60: moderate, Kappa value 0.61–0.80: substantial, Kappa value 0.81–1.00: almost perfect.

*K* Kappa coefficient.

When analyzing the CXR pattern, the inter-observer agreement between the PICU sonologists’ interpretation of CXR results versus the radiologist’s interpretation of CXR results, measured by Cohen’s Kappa, was 0.25 (95% CI 0.18–033) at the baseline visit. For the following visits, the huge amount of missing data for CXR prevented us from carrying out a proper inter-observer agreement analysis. Table 3 shows the agreement results between the radiologist’s and trained physicians’ LUS report for each finding.

**Fig. 2:**
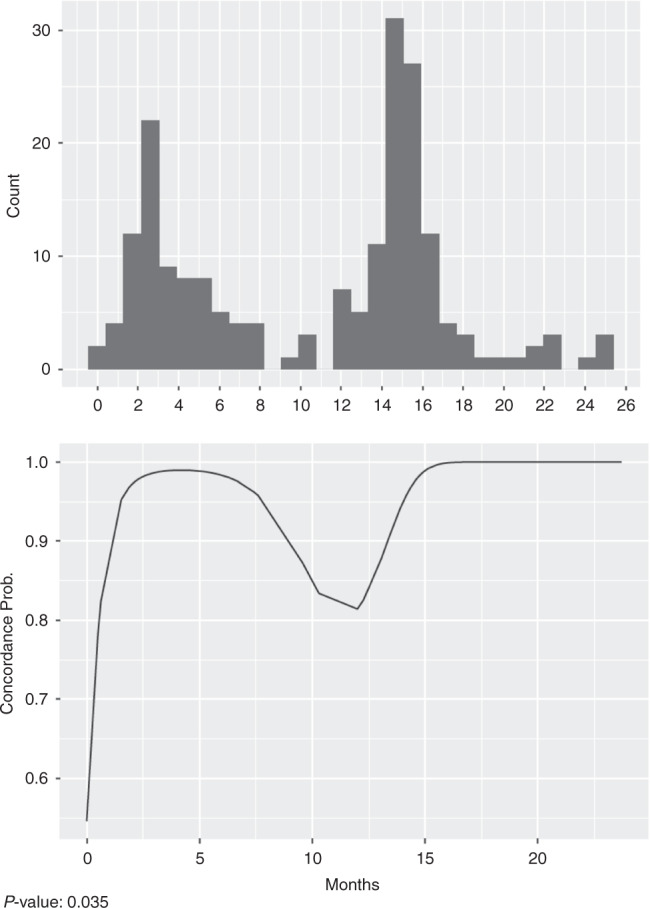
The basic learning curve of aggregate LUS scans. Analysis of the agreement between SRs and PICPs as the study progressed.

The global concordance for interstitial syndrome in the first tercile resulted in 0.44 (0.32–0.55) vs. 0.66 (0.55–0.74) in the third tercile, *p* = 0.096. For the presence of consolidation, this resulted in a concordance of 0.41 (0.14–0.62) vs. 0.94 (0.78–0.98) in the first and third tercile, respectively, *p* = 0.044. For the identification of each consolidation type, the concordance resulted in 0.75 (0.70–0.80) in the first tercile vs. 0.87 (0.81–0.91) in the third tercile, *p* = 0.063. More details are shown in Table 4. A basic learning curve of aggregate LUS scans was obtained to determine whether there was an improved agreement with the radiologists as the study progressed (Fig. 2).

**Table 4:** Global concordance for each tercile, by kappa values calculation.

	Q1	Q2	Q3	*p*-value linear trend
Interstitial syndrome severity				
*K* (95% CI)	0.44 (0.32–0.55)	0.47 (0.31–0.60)	0.66 (0.55–0.74)	0.096
Presence of consolidation				
*K* (95% CI)	0.41 (0.14–0.62)	0.79 (0.60–0.89)	0.94 (0.78–0.98)	0.044
Type of consolidation				
*K* (95% CI)	0.75 (0.70–0.80)	0.84 (0.79–0.87)	0.87 (0.81–0.91)	0.063

*K* kappa value, *Q1* tercile 1, *Q2* tercile 2, *Q3* tercile 3.

## Discussion

This study analyses the results of a training program, which included a large number of LUS on patients with suspected pneumonia. The study followed the steps for implementing a medical diagnostic technique, with both theoretical and practical program components. In the first part, we identified the PICU’s challenges and designed and carried out the training plan. Both the final training program and the test marks yielded excellent results.

For B-line/interstitial syndrome severity, the results showed moderate agreement. However, in our study, most of the non-concordant results involved single B-lines vs. confluent B-lines. Even though Rouby et al. suggest that a B-line/interstitial syndrome severity pattern is easily evaluated with a reduced number of LUS,^31^ it may not be that simple. We would like to draw particular attention to the fact that B-line/interstitial syndrome severity should be the most accurately classified. It would therefore be wise to consider that this classification may have higher inter-observer variability. A greater number of ultrasounds might be recommended to improve this correlation.

When pneumonia is suspected, clinical symptomatology and CXR results are not specific enough to enable a differential diagnosis between viral and bacterial infection, and the final diagnosis might be quite complex.^32^ In this study, the inter-observer agreement analysis showed a substantial agreement for the presence of consolidation in LUS between the physicians trained and the radiologist (close to 0.8), and an almost perfect agreement for the type of consolidation (0.7, 95% CI 0.66–0.73). Therefore, these values lead us to affirm that LUS seems to be a reliable procedure that allows for discrimination between viral and bacterial pneumonia. Compared with other authors, Biagi et al.,^14^ previously examined the effect of experience on LUS accuracy and evaluated the inter-observer agreement between the trained pediatrician and pediatric radiologists sonologists, for the diagnosis of pneumonia in patients with bronchiolitis. They found an excellent inter-observer agreement with a *K* value of 0.93 and concluded that the almost perfect interrater reliability shown between novice and expert users supports the use of LUS as a basic, easy-to-learn sonographic technique.^12,14^ Another study determined the interrater reliability of LUS for detecting pediatric pneumonia compared with chest CT scans, resulting in a *K* of 0.55 (95% CI 0.40–0.70).^33^

Our study demonstrates that developing a training plan is an interesting and important endeavor that allows us to achieve accurate and homogeneous LUS pneumonia diagnoses. By contrast, the results of the inter-observer agreement analysis when assessing the consolidation pattern using CXR showed this technique to be much less accurate, even though CXR is much more widely used.^14,34^ As other authors reported, inter-observer agreement for CXR interpretation results when analyzing the presence or absence of pneumonia proved to be fair to moderate, especially among junior pediatric physicians, which specifically resulted in a *K* of 0.37. Our study also bolsters the results of previous studies, which state that pneumonia shows consistent ultrasound images for children and emphasize that its diagnostic accuracy is beyond that of conventional radiology.^8,9^ Moreover, our results are similar to the studies by Nadimpalli et al.^35^ and Tsung et al.,^36^ in which LUS was used to evaluate bacterial versus viral pneumonia. They found similar values to our results as regards the presence of consolidation on LUS: 0.73 (95 % CI 0.63–0.82) and 0.82 (95% CI 0.79–0.85), respectively.

Pleural effusion is a common condition in critical care patients. LUS may help clinicians not only to visualize pleural effusion, but also to distinguish between the different types. LUS is essential during thoracentesis and chest tube draining, as it increases safety and decreases life-threatening complications.^37^ Some authors described a pleural effusion diagnostic accuracy of 91% for LUS, compared with 56% for physical examination and 33% for X-ray examination.^38^ In our analysis, the results for the presence of pleural effusion showed substantial agreement between the physicians trained and the radiologist (0.78, 95% CI 0.66–0.86). This highlights the importance and benefits of the LUS technique for a better diagnosis and treatment of pleural effusion.

As regards the evaluation of pneumothorax, we must point out that our results are not meaningful due to the few cases of pneumothorax in our study. This would explain why the *K* for the pneumothorax evaluation is close to zero. What could be concluded here is that there is high agreement as regards the presence of pneumothorax, since the number of disagreements between the pediatrician and the radiologist was just one out of three cases. However, if in a particular category the prevalence of the finding is very low, we cannot guarantee that observers really agree in that category. This is the reason why in a qualitative inter-observer agreement study, it is recommended to balance the proportions as much as possible. Obviously, the design of our sample was observational, and this could not be controlled.

As for the basic analysis of the learning curve, for the interstitial syndrome pattern, there seems to be an upward trend in the third tercile. The concordance reaches its maximum values starting at 15 months. When comparing Kappa indexes, the p-value is low but not significant. However, given that the *p-*value is <0.1, the authors interpreted this as a possible improvement during the study. For the identification of the presence of consolidation, there was a clear upward trend. In the last quartile, the concordance is almost perfect and the test is significant. Regarding the analysis of the consolidation type, there was also an upward trend in concordance, but in this case, the results started from higher concordance values than those for the presence of consolidation, so the impact seen is less pronounced. Due to the few cases of pleural effusion and pneumothorax, the permutation test was clearly useless. Other authors^35^ conclude that it is feasible to train clinical practitioners in LUS to diagnose pneumonia and other pulmonary diseases. In light of our results, we could say that the skill of the physicians trained in LUS improved throughout the study. However, it is important to emphasize the importance of continuing with training on and performing LUS, even for a trained physician, because as shown in Fig. 2, when less LUS is done, inter-observer concordance may decrease.

Previously published papers have shown that e-learning programs can be used to teach LUS^39,40^ and the combination of interactive learning concepts and blended activities can boost skills. Moreover, e-learning programs yield similar results to classic classroom-based presentations.^41^ E-learning methods for teaching theoretical knowledge have the advantage of offering flexibility in terms of the time, place, and pace of the learning activity. Having our physicians also be specially trained LUS operators may improve healthcare quality.^2,11,16^ The training program designed in this study, which was based on previous studies conducted in adults,^1,13^ can be followed to teach non-trained physicians how to use LUS, particularly for pneumonia diagnosis.

## Limitations

We acknowledge that this study has some limitations. The main limitation is that it was performed in the PICU of a single hospital. Nevertheless, a large sample of patients was included and a large sample of LUS were performed, which permitted the comparison of homogeneous populations. Moreover, considering the existing literature, when comparing CXR to LUS, it is suggested that both tests are complementary, but that ultimately LUS is the better single test. Using CXR as the reference standard is probably a flawed approach, as the gold standard image test is computed tomography. However, the latter is not practical due to costs and safety nor ethical, considering radiation issues. Another limitation is that the use of ultrasound for the diagnosis of lung infections is an operator-dependent skill, meaning it may lead to a bias in the practical assessment of LUS. Finally, due to the very low prevalence of pneumothorax, the related Kappa results are likely not meaningful.

In conclusion, the implementation of a LUS training program might optimize pneumonia diagnosis in critically ill patients and lead PICU physicians to improve their skills in this field, thus enhancing patient care. There is a high agreement between the physicians trained and the radiologists for the diagnosis of the presence of consolidation and each type of consolidation. These results encourage the use of LUS not only for pneumonia diagnosis but also for discerning bacterial and viral patterns.

Disseminating this work to the scientific community might help other physicians to develop and improve their skills in performing and interpreting LUS.

## Supplementary information

Supplementary Appendix

Supplementary Pre and post training test exam

Supplementary Student satisfaction survey

Supplementary material. Flow diagram
