# Supplementary material for: A training plan to implement lung ultrasound for diagnosing pneumonia in children
Source: Pediatr Res. 2021 Dec 30;92(4):1115–21. doi: 10.1038/s41390-021-01928-2 (PMC9586858; doi:10.1038/s41390-021-01928-2)
Supplement: Supplementary file 1 — Supplementary Appendix [file 41390_2021_1928_MOESM1_ESM.pdf]

Appendix. MATRIX TABLES FOR ALL LUNG ULTRASOUND FINDINGS.

1. Aeration pattern (3x3 matrix tables) for each visit.

Baseline visit, visit 1 (first visit), visit 2 (second visit).

Eco P: lung ultrasound report by pediatrician; Eco R: lung ultrasound report by radiologist.

Classification:

1: A-lines only.

2: focal/single B lines.

3: Confluent B lines.

Table 4: Baseline visit

Eco P/Eco R	1	2	3	Total
1	4	4	0	8
2	6	141	7	154
3	0	12	16	28
Total	10	157	23	190

Table 5: Visit 1

Eco P/Eco R	1	2	3	Total
1	3	4	0	7
2	0	127	10	137
3	0	7	11	18
Total	3	138	21	162

Table 6: Visit 2

Eco P/Eco R	1	2	3	Total
1	4	7	0	11
2	1	100	6	107
3	0	8	3	11
Total	5	115	9	129

Table 7: Kappa Index

visita	kappa	LL95	UL95
0	0.56	0.41	0.69
1	0.55	0.36	0.71
2	0.39	0.15	0.58
Global	0.51	0.43	0.58

Comparison between 3 kappa index-p-value = 0.333

A global kappa index may be given for the three visits. It results in 0.51 (moderate)

The most discrepancies occur between categories 2 and 3.

2. Presence of consolidation.

Baseline visit, visit 1 (first visit), visit 2 (second visit).

Eco P: lung ultrasound report by pediatrician; Eco R: lung ultrasound report by radiologist.

Classification in:

Si: Yes

No: No

Table 8: Baseline visit

Eco P/Eco R	Si	No	Total
Si	170	8	178
No	3	9	12
Total	173	17	190

Table 9: Visit 1

Eco P/Eco R	Si	No	Total
Si	147	5	152
No	1	9	10
Total	148	14	162

Table 10: Visit 2

Eco P/Eco R	Si	No	Total
Si	108	6	114
No	6	9	15
Total	114	15	129

Table 11: Kappa Index

visita	kappa	LL95	UL95
0	0.59	0.37	0.81
1	0.73	0.53	0.94
2	0.55	0.32	0.77
Global	0.67	0.53	0.78

Comparison between 3 kappa index-p-value = 0.827

The hypothesis that the K index is the same for the three visits is not rejected. So that, a global kappa index might be given for the three visits. It results in 0.67 (substantial).

3. Type of consolidation

Baseline visit, visit 1 (first visit), visit 2 (second visit).

Eco P: lung ultrasound report by phyisician; Eco R: lung ultrasound report by radiologist.

Table 16: Baseline visit

Eco P/Eco R	1	2	3	7	Total
1	75	2	0	0	77
2	3	46	4	4	57
3	2	8	32	2	44
7	0	2	1	9	12
Total	80	58	37	15	190

Table 17: Visit 1

Eco P/Eco R	1	2	3	7	Total
1	58	3	1	1	63
2	3	46	4	0	53
3	3	3	27	3	36
7	0	1	0	9	10
Total	64	53	32	13	162

Table 18: Visit 2

Eco P/Eco R	1	2	3	7	Total
1	47	2	1	1	51
2	0	32	1	1	34
3	0	2	25	2	29
7	2	3	1	9	15
Total	49	39	28	13	129

Table 19: Kappa Index

visita	kappa	LL95	UL95
0	0.79	0.71	0.86
1	0.80	0.73	0.88
2	0.83	0.75	0.90
Global	0.82	0.79	0.85

Classification in:

1: lung consolidation with air bronchograms

2: coalescent B-lines with small subpleural consolidations

3: lung consolidation without air bronchogram

7: no consolidation

Comparison between 3 kappa index-p-value = 0.842

The hypothesis that the K index is the same for the three visits is not rejected. So that, a global kappa index might be given for the three visits. It results in 0.82 (almost perfect).

4. Presence of pleural effusion

Baseline visit, visit 1 (first visit), visit 2 (second visit).

dp P: lung ultrasound report by pediatrician; dp R: lung ultrasound report by radiologist.

Classification:

No: No

Si: Yes

Table 24: Baseline visit

dp P/dp R	No	Si	Total
No	167	7	174
Si	3	13	16
Total	170	20	190

Table 25: Visit 1

dp P/dp R	No	Si	Total
No	145	3	148
Si	2	12	14
Total	147	15	162

Table 26: Visit 2

dp P/dp R	No	Si	Total
No	113	9	122
Si	2	5	7
Total	115	14	129

Table 27: Kappa Index

visita	kappa	LL95	UL95
0	0.69	0.52	0.87
1	0.81	0.65	0.97
2	0.44	0.16	0.71
Global	0.78	0.66	0.86

Comparison between 3 kappa index-p-value = 0.161

Comparison between IK for the second visit in relation to the other two – p-value= 0.048 kappa index-p-value

The global kappa index refers to visits 0 and 1. It resulted in 0.78 (substantial).

5. Presence of pneumothorax

Baseline visit, visit 1 (first visit), visit 2 (second visit).

dp P: lung ultrasound report by pediatrician; dp R: lung ultrasound report by radiologist.

Classification:

No: No

Si: Yes

Table 28: Baseline visit

ECO P/ECO R	Si	No	Total
Si	0	0	0
No	0	190	190
Total	0	190	190

Table 29: Visit 1

nt P/nt R	Si	No	Total
Si	1	1	2
No	0	160	160
Total	1	161	162

Table 30: Visit 2

nt P/nt R	Si	No	Total
Si	1	0	1
No	0	128	128
Total	1	128	129

Table 31: Kappa Index

visita	kappa	LL95	UL95
0	0.00	0.00	0.0
1	0.66	0.05	1.0
2	1.00	1.00	1.0
Global	0.15	0.09	0.2

Comparison between 3 kappa index-p-value = 0.242

The hypothesis that the K index is the same for the three visits is not rejected. So that, a global kappa index might be given for the three visits. It results in 0.15 (fair).

Due to the small sample of pneumothorax cases, the K estimate yielded a low value of 0.15. Since the prevalence of pneumothorax in the sample does not represent the prevalence of pneumothorax in the population, the sample is not valid to assess the concordance of pneumothorax since we miss the presence of this finding.
