# Supplementary material for: A training plan to implement lung ultrasound for diagnosing pneumonia in children
Source: Pediatr Res. 2021 Dec 30;92(4):1115–21. doi: 10.1038/s41390-021-01928-2 (PMC9586858; doi:10.1038/s41390-021-01928-2)
Supplement: Supplementary file 2 — Supplementary Pre and post training test exam [file 41390_2021_1928_MOESM2_ESM.pdf]

Pre-training and post-training test exam

1. Regarding reflective surfaces, which of the following is true?

A. Reflective surfaces do not modify the echoes during an ultrasound.
B. Reflective surfaces are the interfaces between two different physical media.
C. Reflective surfaces are independent of the medium's density.
D. Reflective surfaces only reflect echoes and never let them pass through.

2. Which of the following is/are true about the piezoelectric effect?

A. It transforms electrical energy into acoustic energy, and vice-versa.
B. It is caused by the crystals present in the exploration probes.
C. It gives information that is processed in the processing unit and transformed into images on the monitor.
D. All of the above are true.

3. With the Doppler effect:

A. We measure the speed of a moving surface.
B. When colourising the image, red colour shows that the fluid is approaching the probe and blue colour that the fluid is moving away from it.
C. Neither of the above are true.
D. A and B are both true.

4. The probes or transducers can be:

A. Convex or low frequency for superficial tissues.
B. Linear or high frequency for deep tissues.
C. Sectorial with a small, low-frequency contact area that allows high-resolution images to be taken of deep structures.
D. None of the above are correct.

5. The elementary images in ultrasound are:

A. Anechoic images are those with no internal echoes, and they are generated by liquid substances.
B. Hypoechoic images are those that show a higher echogenicity compared to the surrounding tissue (whiter).
C. Hyperechoic images are those that show a lower echogenicity compared to the surrounding tissue (darker grey).
D. All these answers are true.

6. The indicator on the transducer:

A. Helps us orient ourselves.
B. Should be positioned preferably facing right or upwards.
C. Will be shown on the left side of the monitor.
D. All of the above are true.

7. Pulmonary ultrasound:

A. Cannot be used because the lungs are filled with air and you cannot see them.
B. Could help in the diagnosis of different pathologies.
C. Makes the presence of pleural effusion easily visible.
D. B and C are correct.

8. Which of these images are pathological on a chest ultrasound?

A. Bat sign.
B. A-lines.
C. Lung sliding.
D. More than 8 confluent B-lines.

9. If we see any of these signs when performing lung ultrasound, we can be sure of the presence of pneumothorax:

A. Jellyfish sign.
B. Lung sliding.
C. B-lines in any number.
D. Lung point.

10. Pleural effusion could be seen as:

A. An anechoic or hypoechoic entity, even though there may be isoechoic or hyperechoic tracts.
B. A phrenic line.
C. Neither of the above is correct.
D. Both A and B are correct.

11. The presence of multiple B-lines or comet tails indicates:

A. Increased air in the lungs.
B. Increased fluid in the lungs.
C. Potential acute respiratory disease syndrome (ARDS).
D. Answers B and C are correct.

12. In a patient with pneumothorax, which of the following signs would we NEVER observe?

A. Lung sliding.
B. Lung point.
C. Stratosphere sign (M-mode).
D. B-lines in the area without pneumothorax.

13. Which of the following definitions is NOT correct:

A. In a patient who is not breathing, we will not be able to visualise lung sliding.

B. B-lines are an artifact present in the normal lung.
C. The seashore sign is present in M-mode in normal lung.
D. The stratosphere sign is present in M-mode in normal lung.

14. Regarding B-lines, which of the following definitions is correct:

A. They emerge from the pleural line.
B. B-lines are a sign of perialveolar oedema.
C. B-lines are absent when there is pneumothorax.
D. All of the above are correct.

15. Regarding A-lines, which of the following definitions is NOT correct:

A. They are parallel to the pleural line.
B. They are absent when there is pneumothorax.
C. A-lines may overlap with B-lines.
D. A-lines might be seen in patients with ARDS.
