# Supplementary material for: A training plan to implement lung ultrasound for diagnosing pneumonia in children
Source: Pediatr Res. 2021 Dec 30;92(4):1115–21. doi: 10.1038/s41390-021-01928-2 (PMC9586858; doi:10.1038/s41390-021-01928-2)
Supplement: Supplementary file 3 — Supplementary Student satisfaction survey [file 41390_2021_1928_MOESM3_ESM.pdf]

Student satisfaction survey

A - ORGANISATION

1. Was the organisation of the course appropriate?
2. Are you satisfied with the quality of the documentation provided?
3. Were the organisational aspects respected?
4. Were the classrooms well-suited to the training?

B - METHODOLOGY

1. Was the structure of the course content suitable?
2. Was the teaching methodology used appropriate?
3. Was there enough time to adequately cover the content?
4. Was it useful to acquire new knowledge?
5. Was it useful to acquire new skills?
6. Was it useful to incorporate new attitudes?

C - APPLICABILITY

1. Is there a relationship between the content of the course and your current work?
2. Was this course useful for you in your work?
3. Were you able to improve your professional development with this course?

D - TEACHERS

1. Was a climate of trust created?
2. Did you master the topic at hand?
3. Did they explain everything clearly?
4. Did they arouse your interest?
5. Did they encourage your participation?
6. Did they listen to the students with interest?
7. Did they facilitate the exchange of experiences?
8. Did they constructively correct students' mistakes?

E - QUALITY OF THE THEORETICAL CLASSES

1. Generalities
2. Normal lung pattern
3. Consolidation pattern
4. Interstitial syndrome
5. Pleural effusion
6. Pneumothorax

F - QUALITY OF THE PRACTICAL CLASSES

Item	0 Very poor	1 Poor	2 Fair	3 Good	4 Very good	5 Excellent
**A - ORGANISATION**						
Was the organisation of the course appropriate?						
Are you satisfied with the quality of the documentation provided?						
Were the organisational aspects respected?						
Were the classrooms well-suited to the training?						
**B - METHODOLOGY**						
Was the structure of the course content suitable?						
Was the teaching methodology used appropriate?						
Was there enough time to adequately cover the content?						
Was it useful to acquire new knowledge?						
Was it useful to acquire new skills?						
Was it useful to incorporate new attitudes?						
**C - APPLICABILITY**						
Is there a relationship between the content of the course and your current work?						
Was this course useful for you in your work?						
Were you able to improve your professional development with this course?						
**D - TEACHERS**						
Was a climate of trust created?						
Did you master the topic at hand?						
Did they explain everything clearly?						
Did they arouse your interest?						
Did they encourage your participation?						
Did they listen to the students with interest?						
Did they facilitate the exchange of experiences?						
Did they constructively correct students' mistakes?						
**E - QUALITY OF THE THEORETICAL CLASSES**						
Generalities						
Normal lung pattern						
Consolidation pattern						
Interstitial syndrome						
Pleural effusion						
Pneumothorax						
**F - QUALITY OF THE PRACTICAL CLASSES**						
