# Supplementary figures and images for: A training plan to implement lung ultrasound for diagnosing pneumonia in children
Source: Pediatr Res. 2021 Dec 30;92(4):1115–21. doi: 10.1038/s41390-021-01928-2 (PMC9586858; doi:10.1038/s41390-021-01928-2)

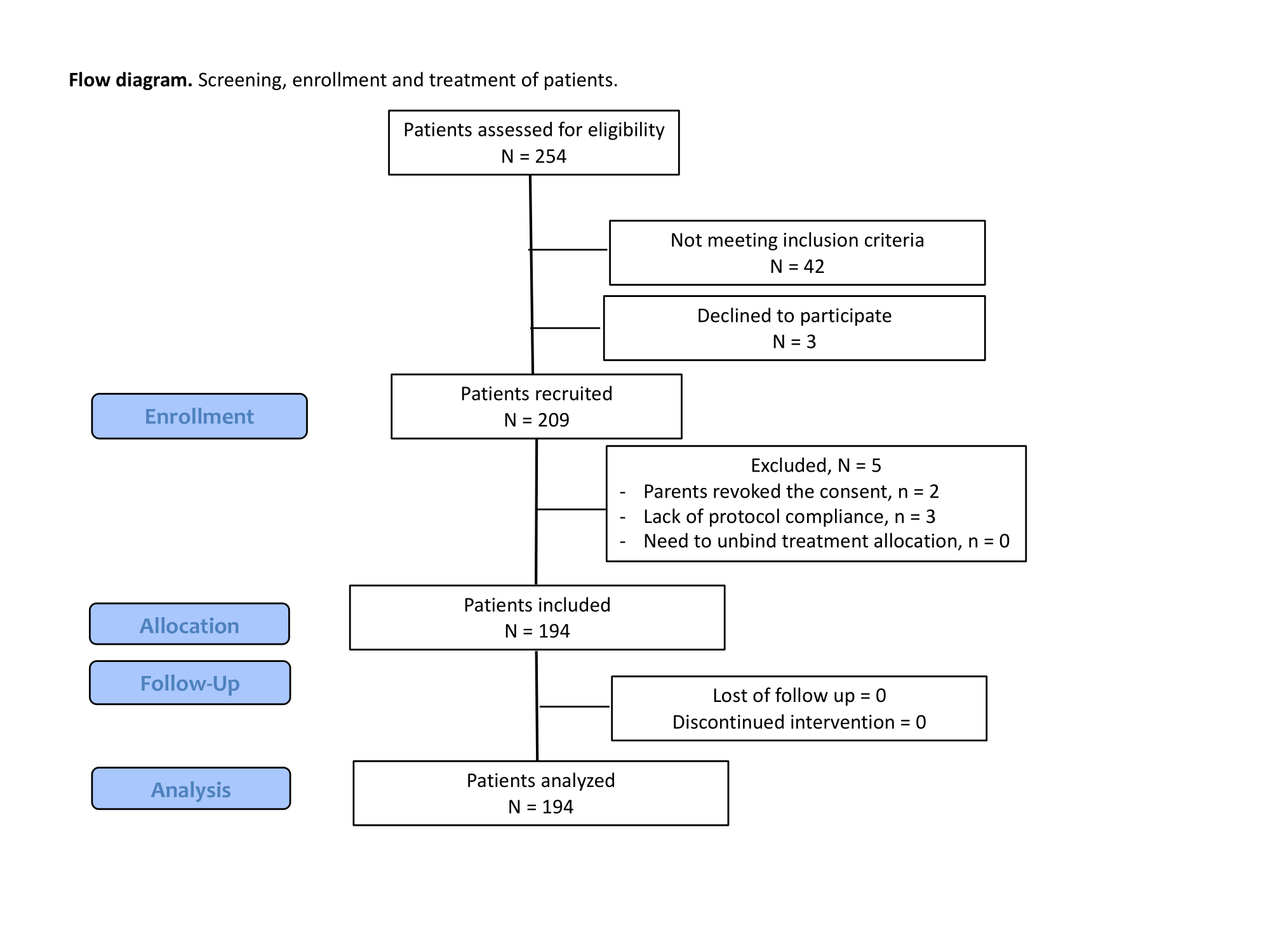

Supplement: Supplementary file 4 — Supplementary material. Flow diagram [file 41390_2021_1928_MOESM4_ESM.tiff]
